# Analysis on process of temporal and spatial evolution of urban built-up area expansion in the Yellow River Basin

**DOI:** 10.1371/journal.pone.0270370

**Published:** 2022-07-08

**Authors:** Lin Fan, Baifa Zhang, Yihang Wang, Wei Zhao, Shuai Dong

**Affiliations:** 1 College of Geography and Environmental Science, Henan University, Kaifeng, Henan, China; 2 Key Research Institute of Yellow River Civilization and Sustainable Development Collaborative Innovation Center on Yellow River Civilization, Kaifeng, Henan, China; 3 National Demonstration Center for Environment and Planning, Henan University, Kaifeng, Henan, China; Zhongnan University of Economics and Law, CHINA

## Abstract

Urban spatial expansion is known as an important indicator of urbanization. In order to provide a reference for urban spatial expansion in the future high-quality development strategy of the Yellow River Basin (YB) cities in China, it is necessary to identify and calculate urban spatial expansion patterns. For this reason, we provide a "Spatiotemporal pattern-Center of gravity migrationt-Expansion pattern" solution to identify and calculate urban spatial expansion patterns in the YB. More specifically, 78 prefecture-level cities in the YB were selected as the subjects of the study, using the Defense Meteorological Satellite Program/Operational Line Scan System (DMSP/OLS) and the National Polarimetric Partnership/Visible Infrared Imaging Radiometer Suite (NPP/VIIRS) nighttime light data (NTL), together with the center of gravity shift and common edge detection models, to identify the YB urban expansion patterns from 2000–2018. The results suggest that: (1) on the spatial pattern, there is a obvious difference in the expansion intensity and growth rate of the urban built-up (UB) areas of cities in the upper and middle reaches of YB. In addition, there are also certain differences between the expansion patterns of provincial capital cities and non-capital cities; (2) The UB areas of YB has steadily expand from 3,500 km^2^ in 2000 to 10,600 km^2^ in 2018, amongst which the expansion of provincial capital cities is the most obvious 1919 km^2^; (3) Interestingly it is also discovered that urban expansion in Qinghai Province, the sourceland of the YB, takes place in a diffuse way, with the shifting of the centre of gravity for four types of total area, net increase in area, rate of growth and intensity of expansion followed a "northwest to southeast" tendency of development.

## Introduction

During China’s reform and opening up, the urbanization rate in China has increased from 17.92% in 1978 to 63.89% in 2020. Rapid urbanization has transformed a large number of rural people into urban residents, and the population has gathered in cities, which has promoted rapid economic development, and the UB area has expanded rapidly as the urbanization process has accelerated. Over the past four decades, the area of UB areas in China has increased by 8.1 times and continues to grow (http://www.stats.gov.cn/tjsj/ndsj/). Urban spatial expansion is an important symbol of urbanization, and the study of UB area expansion is of great significance to identify the urbanization process and judge the urban development stage. Simultaneously, the development of remote sensing and geoinformation technology has led to the innovation of urban expansion detection technology, and the emergence of NTL data has, to a certain extent, made it possible for urban studies to conduct multi-scale, large-scale, and long-time period urban expansion detection [1,2], and it also has the advantages of large coverage, fast timeliness, and easy accessibility, which can be widely used in multi-scale and long-time period urban problem studies [3].

The research on NTL covers many fields, including urban economic development [4], population density estimation [5], disaster [6], fishery [7,8], carbon emission [9], energy consumption [10]. With the rapid advancement of urbanization in China, various problems caused by urban spatial expansion have become increasingly prominent, along with a large number of study results have emerged [11]. NTL have been widely used in the study of urban spatial expansion [12–17], and the dynamic study of urban spatial pattern has gradually become a study hotspot [5,14,18,19] compared the expansion rate and intensity of urban construction land from different spatial scales, so as to explore the spatial and temporal evolution pattern of urban expansion. Liu Xiaoping (2009) and Liu Zhifeng(2012) found that most of the urban construction land area showed an expansion trend [20,21], and the expansion intensity was different in time and space [22,23] used remote sensing image data to compare and analyze the spatial and temporal characteristics of land expansion in 10 cities in Russia, and revealed the evolution characteristics of urban expansion pattern. Qingling Zhang (2011) combined DMSP/OLS data with population and land use data to analyze the urban expansion in China, the United States, India and Japan from 1992 to 2000 [24]. Jiang, Sun, & Zheng (2019) used NPP/VIIRS data to analyze the urban expansion of Xiamen, Quanzhou and Zhangzhou in Fujian Province from 2013 to 2017 [25]. It is believed that there is a strong correlation between night light brightness value and human social and economic activities. Based on NTL, Wan Yi (2019) studied the spatial pattern status and urban expansion process of various cities in Henan Province from the aspects of urban focuses coordinates, urban focuses offset distance and urban focuses offset speed [26]. Some ones studied and analyzed the change and trend of urban spatial structure in Chongqing based on NPP NTL data [27–30]. Others analyzed various data changes in cities, and study the temporal and spatial evolution pattern of urban expansion by using urban expansion speed index, urban expansion intensity index, urban expansion amplitude index, elasticity coefficient of urban construction land expansion and other indicators [2,12,31,32]. By combing the previous studies, it is found that most of the existing studies on the expansion of built-up areas described the evolution of spatio-temporal pattern, and the exploration of urban expansion mode is relatively lacking. Scholars at home and abroad have few studies on the expansion speed, shift of focuses and expansion mode of UB areas in the YB according to NTL.

Existing relevant studies mainly focus on studying individual cities, urban agglomerations and provincial cities as study areas, and less often explore urban expansion using natural watersheds as study units. In addition, on September 18, 2019, the ecological protection and high-quality development of the YB was elevated to a major Chinese national-level development strategy. Therefore, this study explores the spatial and temporal characteristics of urban expansion in the YB from the perspective of urban expansion in the basin as a study unit, aimed at revealing the urbanization development process in the YB and providing some reference for the urban expansion as well as urbanization process in other basins.

Based on the above background, this paper takes 78 prefecture-level cities in the YB as the study objects, selecting DMSP/OLS NTL in 2000, 2005 and 2010 and NPP/VIIRS night light remote sensing image data in 2015 and 2018 as data sources, so as to extract the UB areas in the YB in five periods. Then, the urban expansion measurement model, shift of focuses model and common edge detection method are used to analyze the built-up area of the YB from three aspects of "Spatiotemporal pattern-Center of gravity migrationt-Expansion pattern" in 2000–2005, 2005–2010, 2010–2015 and 2015–2018. It revealed the expansion intensity, expansion rate, expansion trend and expansion mode of UB areas in the YB, and described the temporal and spatial variation characteristics of regional development from a macro perspective, providing reference for the sustainable and high-quality development of urban economy in the YB.

## Study area and data sources

### Study area

**Fig 1:**
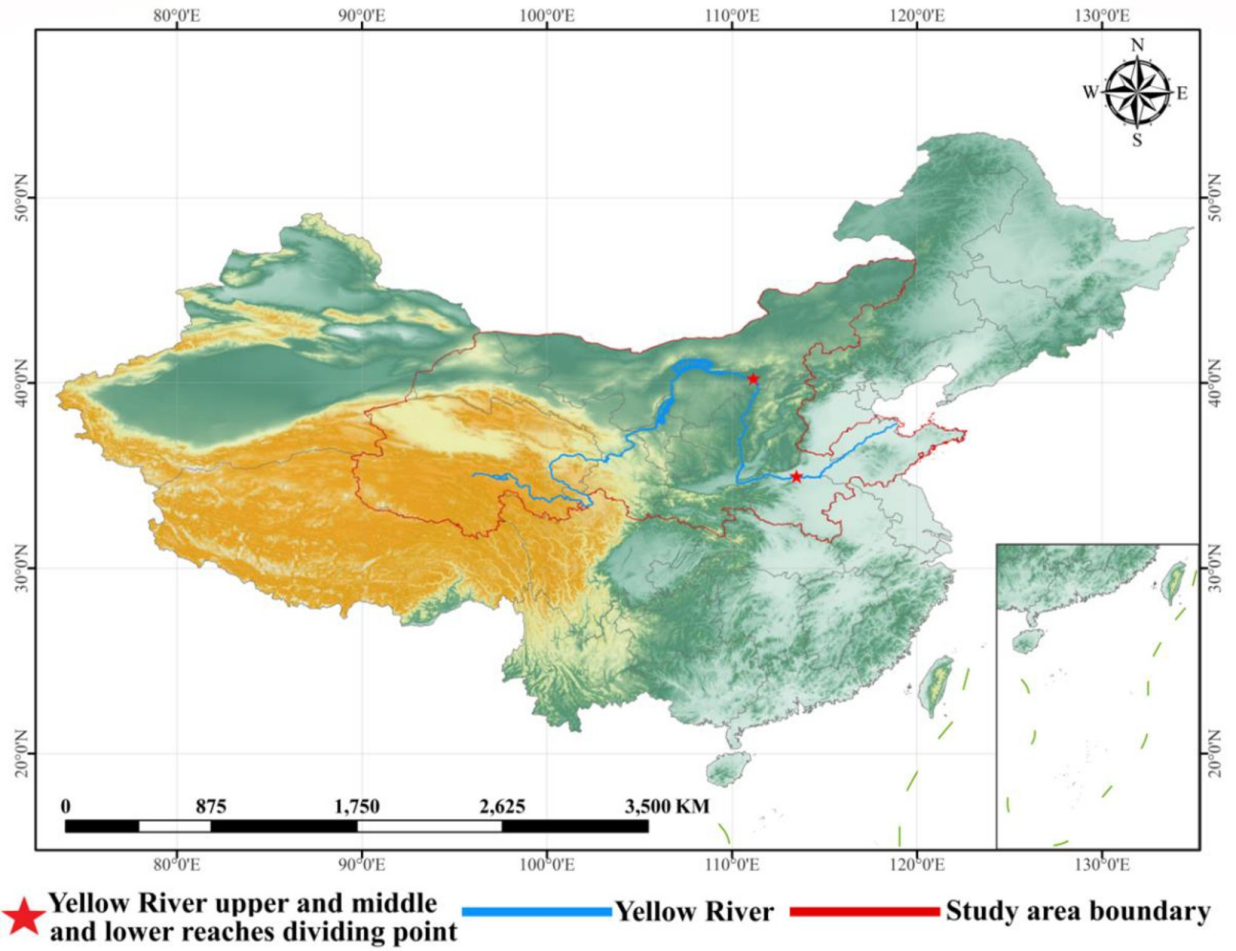
Study area. The origin of the Yellow River to Hekou town in Inner Mongolia is the upstream, Hekou town to Taohuayu near Zhengzhou is the midstream, and below Taohuayu to the mouth of the Yellow River into the sea is the downstream; Location of the Yellow River; Study area boundary.

The YB is located between 32° and 42° north latitude, 95° and 119° east longitude, originated in the northern foothills of the Bayankara Mountains on the Qinghai-Tibet Plateau at an altitude of 4500m in the Yoguzongli basin, flowing through nine provinces of Qinghai, Sichuan, Gansu, Ningxia, Inner Mongolia, Shaanxi, Shanxi, Henan and Shandong, from Heyuan to the town of Hekou in Inner Mongolia for the upper reaches, Hekou to Taoyu near Zhengzhou for the middle reaches, Taoyu below to the mouth of the Yellow River is downstream Fig 1. The topography of the YB varies greatly from west to east, spanning the Qinghai-Tibet Plateau, the Inner Mongolia Plateau, the Loess Plateau and the North China Plain. Under the influence of resource endowment, development base, and national regional development policies, the economic development within the YB is highly uneven, with varying levels of economic development among cities in the upper and middle reaches [33]. Considering other factors such as the completeness and comprehensiveness of the study area, as well as the availability of data, this study finally defines the population of this study as cities other than Sichuan Province and Qinghai Province.

### Data sources

The data sources of the study are as follows:

**(1)** The 250m Digital Terrain Elevation Model (DEM) remote sensing image data of China and the administrative vector data of YB are derived from the 1:250,000 China Basic Geographic Data provided by the Resource and Environmental Science Data Center of Chinese Academy of Sciences (https://www.resdc.cn/);
**(2)** DMSP night stable light image data in 2000, 2005 and 2010 are provided by NGDC website with a resolution of 1 km. NPP/VIIRS night light remote sensing data in 2015 and 2018 are provided by the National Oceanic and Atmospheric Administration (https://www.noaa.gov/) with a resolution of 500 meters;
**(3)** The built-up area used to verify the extraction accuracy comes from China Urban Statistical Yearbook (http://www.stats.gov.cn/tjsj/ndsj/).

## Methods and processing

### Selection of optimal threshold for statistical method

The key to the method of extracting UB area from NTL is to obtain the optimal threshold, and then segment the light data according to the threshold, and finally identify the UB area. At present, there are four main methods to obtain threshold: **(1)** Empirical method for threshold [34–36]; **(2)** Mutation detection method [32]; **(3)** Statistical data comparison method [25]; **(4)** Auxiliary image comparison method [15].

According Shu Song (2011) comparative analysis of the four methods above, it believes that the same gray segmentation threshold does not have universality for NTL of different years, and the formulation of the process with strong convenience and high accuracy can be realized by using the statistical method [37]. Therefore, this paper obtained the optimal threshold by using statistical method.

### Measures of urban expansion

This paper selects three indexes: expansion area(*ΔA*), growth rate(*V*) and expansion intensity(*R*) to quantify the temporal and spatial variation characteristics of urban expansion; Expanded area indicates the growth of urban area in a period of time; The growth rate indicates the expansion rate of UB areas in a period of time; Expansion intensity indicates the average annual urban area growth rate in a certain period of time, that is, relative expansion [31], they are defined as:

$$\Delta A=A_{b}-A_{a}$$ (1)

$$V=\left(\sqrt[{T}]{\frac{A_{b}}{A_{a}}}-1\right)\times 100\%$$ (2)

$$R=\frac{A_{b}-A_{a}}{TLA}\times \frac{1}{T}\times 100$$ (3)

In which: *A*_*a*_ and *A*_*b*_ refer to the area of UB areas in the initial and final stages respectively; *TLA* is the total land area and *T* is the time interval.

### Shift of focuses

UB area extracted from DMSP/OLS data is composed of pixels corresponding to different gray values. Urban focus is the most representative measurement index to describe urban spatial distribution, which can better reflect the average position of urban distribution. In this study, the standard deviation ellipse in ArcGIS software is used to calculate the barycentric coordinates, which is used to reveal the spatial distribution characteristics of geographical objects on the overall level and reflect the trend direction of geographical objects. The expression is as follows:

$$\bar{X}=\sum_{i=1}^{m}DN_{i}X_{i}/\sum_{i=1}^{m}DN_{i}$$ (4)

$$\bar{Y}=\sum_{i=1}^{m}DN_{i}Y_{i}/\sum_{i=1}^{m}DN_{i}$$ (5)

In which:($\bar{X},\;\bar{Y}$) is the center coordinate of the *i* pixel; *m* is the number of pixels; *DN*_*i*_ is the gray value corresponding to the *i* pixel.

### Common edge detection method

The common edge detection method is used to determine the urban expansion mode according to the ratio of the public boundary length of land patches in newly built-up areas and current built-up areas of cities to the total side length of land in newly built-up areas of cities, and its division basis is [6,38,39]:

S=L/P (6)

In which: *S* is the index of urban expansion mode; *L* is the length of common edge of land used for newly added UB areas and existing UB areas; *P* is the total perimeter of the land for the newly built-up area of the city. When *S*≥0.5, it is filled expansion; When 0<*S*<0.5, it is spreading type expansion; When *S* = 0, it means that there is no common edge between the land used in the existing built-up area of the city and the land used in the newly added built-up area of the city, which is an enclave expansion.

### Data processing

Data processing mainly includes DMSP light data processing, NPP light data processing and the fusion of the two kinds of light data. Because the NTL come from DMSP and NPP data sources respectively, it is necessary to correct and fit the two data before using them. Firstly, the two data reference systems are defined as WGS-84 coordinate system, and the projection coordinates are unified as Asia Lambert Conformal Conic. Then, the two kinds of data are corrected respectively, and the saturation correction, multi-sensor correction and continuity correction of DMSP are carried out with reference to the existing literature [20,28,40], and then the DMSP lighting data from 1992 to 2013 are obtained; NPP data needs to be corrected through the steps of "annual average image synthesis, negative value elimination, unstable light source and background noise processing, extremely high value processing and continuity correction" [18], so as to obtain NPP lighting data from 2012 to 2018; On this basis, the comparable and continuous lighting data for 27 years from 1992 to 2018 are finally obtained according to the overlapping years of the two kinds of data [29].

### Evaluation of extraction accuracy of built-up area

ArcGIS is used to extract the UB area of 78 prefecture-level cities in the YB. By selecting the optimal threshold, the UB area with the smallest relative error is extracted, and the final UB area is shown in **Table 1**. Comparing the UB area extracted from lighting data with the UB area obtained from China Urban Statistical Yearbook, the relative error of the extracted results is obtained. During the study period, the relative errors of UB areas in the YB from 2000 to 2018 were controlled within 4%. In 2000, 2005 and 2010, the relative error of UB area was controlled within 2.03%, 2.96% and 3.55%, respectively, among which the extraction accuracy in 2015 and 2018 was higher, and the relative error of UB area was controlled within 1.96% and 0.78%, respectively. The relative error of all extraction results was at a low level and the extraction accuracy was high.

**Table 1:** Extraction accuracy assessment of UB in seven provinces of the YB.

Year	Indicators(km^2^)	Gansu Province	Ningxia Province	Inner Mongolia	Shaanxi Province	Shanxi Province	Henan Province	Shandong Province	Total watershed
2000	EA/km^2^	392	127	331	429	492	725	1047	3543
	SA/km^2^	399	129	335	425	486	740	1029	3543
	Error/%	1.75%	1.55%	1.19%	0.94%	1.23%	2.03%	1.75%	0.00%
2005	EA/km^2^	465	240	448	558	591	1197	1759	5258
	SA/km^2^	471	234	450	554	574	1207	1778	5268
	Error/%	1.27%	2.56%	0.44%	0.72%	2.96%	0.83%	1.07%	0.19%
2010	EA/km^2^	580	321	610	769	726	1569	2485	7060
	SA/km^2^	573	310	598	783	720	1547	2450	6981
	Error/%	1.22%	3.55%	2.01%	1.79%	0.83%	1.42%	1.43%	1.13%
2015	EA/km^2^	711	400	752	1008	898	1914	3451	9134
	SA/km^2^	703	408	745	1006	900	1913	3398	9073
	Error/%	1.14%	1.96%	0.94%	0.20%	0.22%	0.05%	1.56%	0.67%
2018	EA/km^2^	754	436	766	1258	944	2255	4202	10615
	SA/km^2^	753	437	772	1253	951	2266	4228	10660
	Error/%	0.13%	0.23%	0.78%	0.40%	0.74%	0.49%	0.61%	0.42%

Extraction area (EA), statistical area (SA) value and error of UB area in YB.

At the same time, from the whole watershed, the error is controlled below 1.50%, and the errors in five time periods are 0.00%, 0.19%, 1.13%, 0.67% and 0.42%, respectively. The extraction accuracy is high, which proves that it is ideal to extract lighting data from built-up areas by statistical method.

## Result analysis

### Analysis of urban expansion and evolution

**Fig 2:**
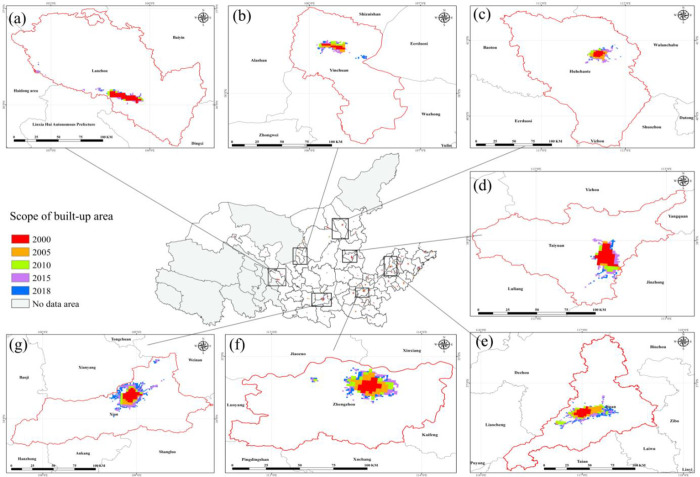
Extraction results of UB areas in the YB from 2000 to 2018. (a) Lanzhou City, capital of Gansu Province (b) Yinchuan City, capital of Ningxia Hui Autonomous Region (c) Hohhot City, capital of Inner Mongolia Autonomous Region (d) Taiyuan City, capital of Shanxi Province (e) Jinan City, capital of Shandong Province (f) Zhengzhou City, capital of Henan Province (g) Xi’an City, capital of Shaanxi Province.

According to DMSP/OLS NTL in 2000, 2005 and 2010 and NPP/VIIRS NTL in 2015 and 2018, the optimal threshold is obtained by means of statistical method, and the UB area of 78 prefecture-level cities in the YB in 2000, 2005, 2010, 2015 and 2018 is extracted according to the optimal threshold. It can be seen from **Fig 2** that the UB areas in the YB are expanding as a whole, especially cities in the lower reaches and provincial capitals are expanding more rapidly, and there are significant differences between the upper, middle and lower reaches, provincial capitals and non-provincial capitals.

### Provincial built-up area expansion analysis

From the provincial perspective, the built-up area of each province increased significantly as shown in Table 1. 19 years from 2000 to 2018, the total built-up area of seven provinces, Gansu, Ningxia, Inner Mongolia, Shaanxi, Shanxi, Henan, and Shandong, increased from 392km², 127km², 331km², 429km², 492km², 725km², and 1047km² in 2000 to 754km², 436km², 766km², 1258km², 944km², 2255km² and 4202km² in 2018, with the built-up areas of Shaanxi, Henan and Shandong provinces expanding rapidly, with a net increase in area reaching 829km², 1530km² and 3155km², respectively.

**Fig 3:**
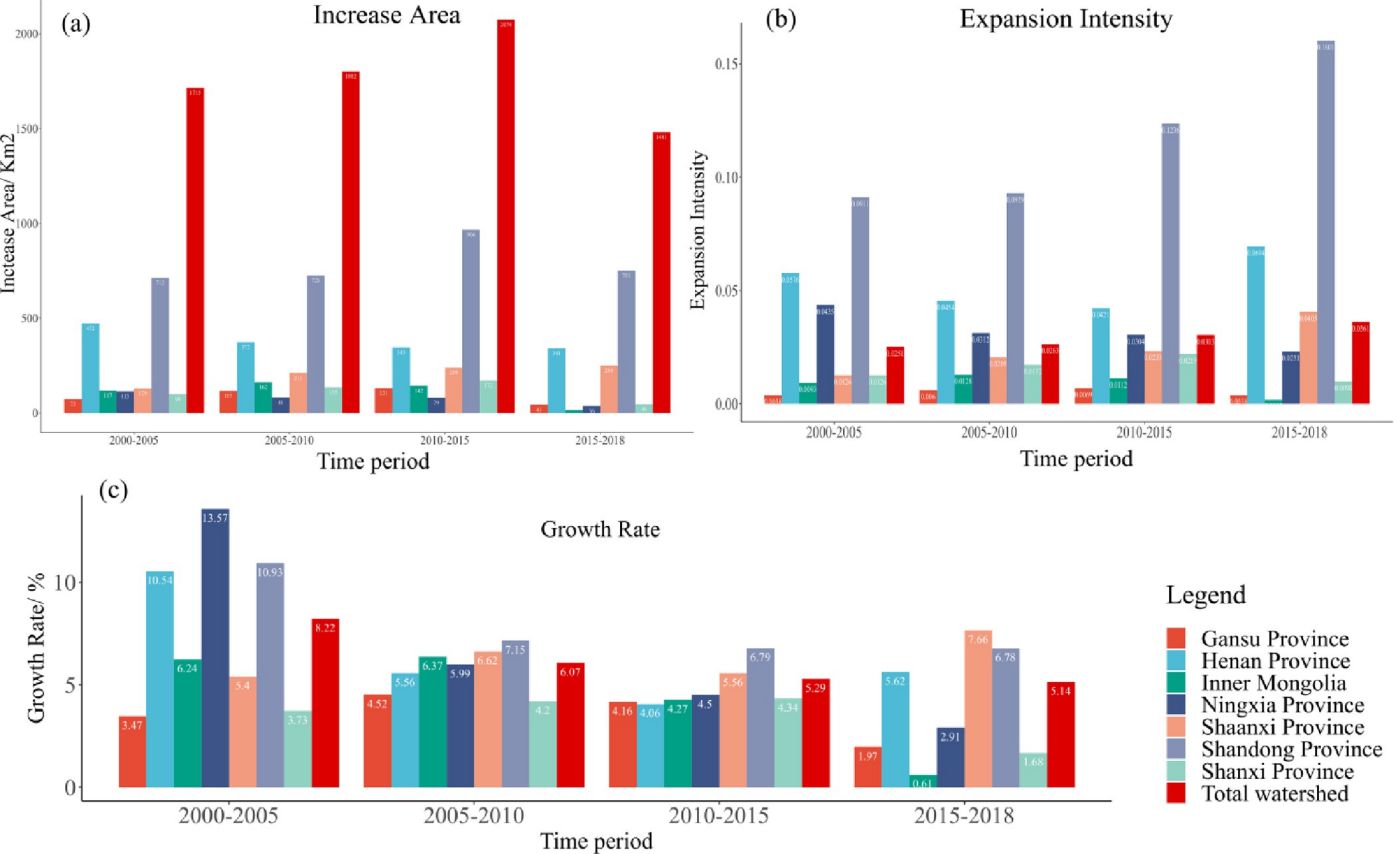
Provincial built-up area expansion area. (a) Change of UB area in the YB, (b) Change of expansion intensity of UB area in the YB, (c) Growth rate of UB area in the YB.

Statistics on the increase of UB area in each province of the study area are shown in Fig 3(A). The net increase of UB area in the YB during the four study time periods from 2000 to 2005, 2005 to 2010, 2010 to 2015, and 2015 to 2018 are 1715 km2, 1802 km2, 2074 km2, and 1481 km2, respectively, indicating that During the period from 2000 to 2018, the UB area of the YB has been growing and the urban space has been expanding; secondly, the expansion intensity of the YB has changed during the four time periods as shown in Fig 3(B), which are 0.0251, 0.0263, 0.0303 and 0.0361, respectively, and the expansion intensity has gradually increased, indicating that the cities are on a continuous expansion trend. The growth rate of built-up area is shown in Fig 3(C), and the growth rates are 8.22%, 6.07%, 5.29% and 5.14% in four time periods, which are fast and show the trend of "gradually decreasing", indicating that the UB area of the YB expands rapidly in the initial years of the study, and then slows down. However, the expansion rate slows down afterwards.

From the urban expansion intensity of the YB in Fig 3(B), during the 19 years from 2000 to 2018, Gansu Province was in a low expansion state, and the expansion intensity of Ningxia, Inner Mongolia and Shanxi provinces decreased from 0.0435, 0.0093 and 0.0126 to 0.0231, 0.0018 and 0.0098, respectively, and the expansion intensity weakened, while the built-up areas of Shaanxi, Henan and Shandong provinces The expansion intensity of built-up areas in Shaanxi, Henan and Shandong provinces increased from 0.0126, 0.0576 and 0.0911 to 0.0405, 0.0694 and 0.1601, indicating that the expansion intensity of built-up areas in these three provinces is high and urban development is strong and still in a rapid development stage.

From the growth rate of the built-up areas in the YB in Fig 3(C), the growth rate of the built-up areas in Ningxia, Henan, and Shandong provinces were the fastest during the initial period of 2000–2005, with 13.57%, 10.54%, and 10.93%, respectively, in the four study time periods, and the growth rate of the three provinces decreased to 2.91%, 5.62%, and 6.78% from 2015 to 2018, indicating that the development of built-up areas gradually shifted from "rapid expansion" to "low and stable expansion". The growth rates of built-up areas in Gansu, Inner Mongolia, and Shanxi provinces fluctuate and decrease, with the growth rates of 3.47%, 6.24%, and 3.73% between 2000 and 2005, respectively, and increase to 4.52%, 6.37%, and 4.20% between 2005 and 2010, respectively, and decrease to 1.97%, 0.61%, and 0.61% between 2010 and 2018, respectively. 1.97%, 0.61% and 1.68%, gradually shifting to a low expansion trend, and the growth rate of the built-up area in Shaanxi Province fluctuates from 5.40% to 7.66%. Among them, Shaanxi, Henan and Shandong growth rates are still in the leading position among the provinces along the Yellow River.

### Cities built-up area expansion analysis

**Fig 4:**
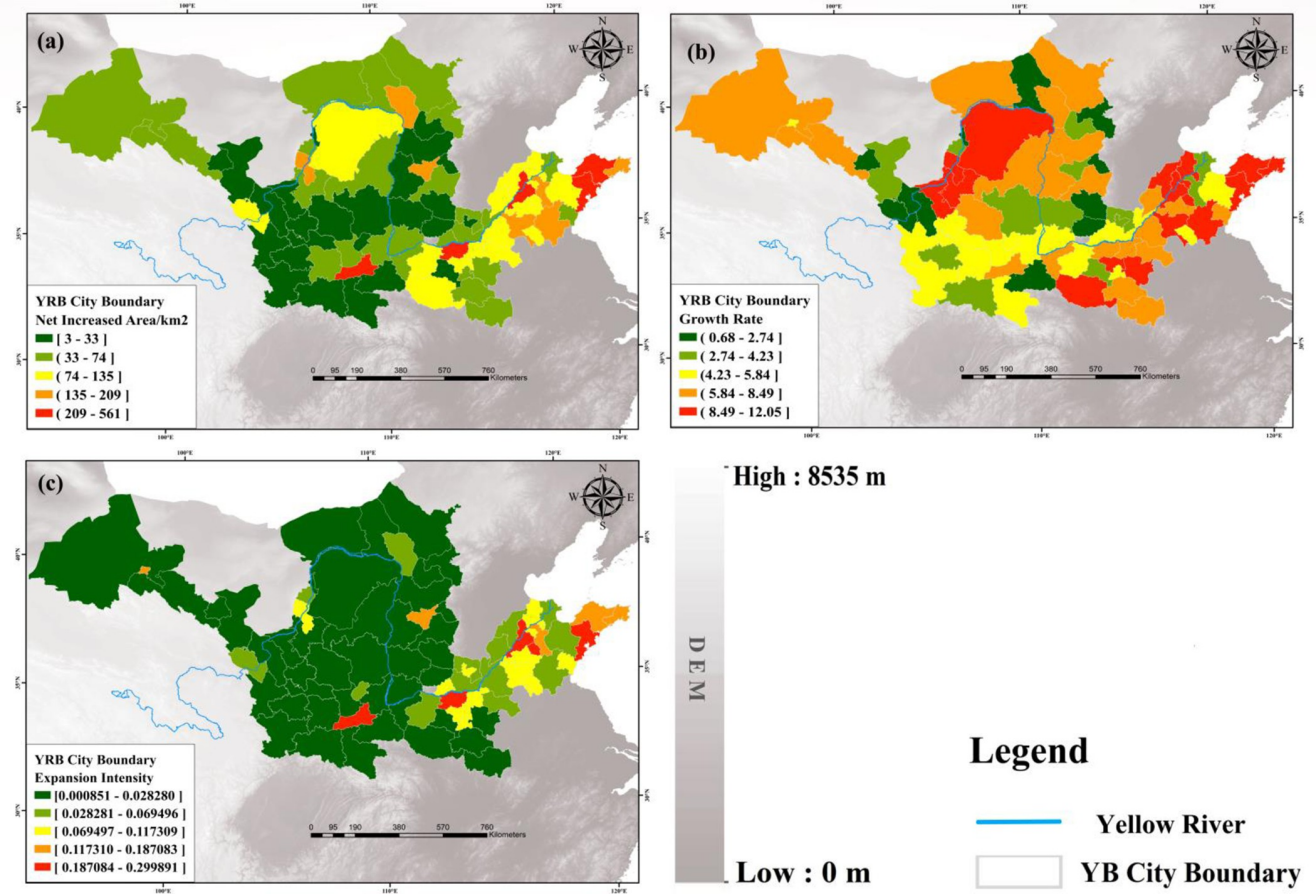
Cities built-up area expansion analysis. **(a)** Distribution of net increased area, **(b)** Growth rate of UB areas and **(c)** Expansion intensity in the YB from 2000 to 2018.

The net increase in built-up area, growth rate and expansion intensity of 78 cities in the YB are shown in Fig 4. From the perspective of the whole basin, there are obvious differences in urban expansion in the upper and middle and lower reaches of the YB, with large net increase in area, high growth rate and strong expansion intensity in the lower reaches. Apart from Shaanxi Province, the middle and upper reaches have a smaller net increase in area, lower growth rate, and weaker expansion intensity compared to the downstream areas. Hence, the study results suggest that the downstream areas have a large UB area expansion and rapid urban development, while the middle reaches have a medium to slow urban development.

From the intra-city perspective, there are large differences between provincial capitals and non-capital cities in terms of net growth area, growth rate and expansion intensity, with large net growth area, high growth rate and strong expansion intensity in provincial capitals and smaller net growth area, lower growth rate and weaker expansion intensity in non-capital cities. Especially in the two indicators of net growth area and expansion intensity, the upper, middle and lower reaches show obvious differences, with large net growth area and strong expansion intensity in the lower reaches, and large differences between provincial capital cities and non-capital cities, with the most representative provincial capital cities led by Jinan, Zhengzhou and Xi’an having large net growth area and strong expansion intensity. As for example, the average net increase in area and expansion intensity in Henan Province during the study period are 90 km^2^ and 0.0668, respectively, while the net increase in area and expansion intensity in Zhengzhou, the provincial capital city, are as high as 406 km^2^ and 0.2999, respectively. Additionally, provincial capital cities such as Jinan and Xi’an, which are similar to Zhengzhou, are much higher than the province’s average in terms of net increase in area and expansion intensity, with Jinan and Xi’an having a high net increase in area of 418 km^2^ and 516 km^2^, respectively, and an expansion intensity of 0.2905 and 0.2838, respectively. According to the Hu Huanyong line division, divided into two parts, the population size in the west is smaller, and from the net increase in area of urban built-up area expansion, except for Erdos, Hohhot, Yinchuan and Lanzhou, the overall net increase in area in the west is small. In terms of expansion intensity, the expansion intensity of Jiayuguan and Yinchuan is higher than that of other regions in the west, and the overall expansion intensity is weaker; while the population size of the eastern region is larger, and the net increase in area of Xi’an, Zhengzhou, Jinan and Qingdao is relatively larger and the expansion intensity is stronger, and in terms of growth rate, the difference in growth rate between the east and west is relatively low. From the division of the urban agglomerations in the YB, the Hubao-Egyu urban agglomeration in the upstream region, as the economic development center of the upstream region, has a smaller area for each city in the starting year, so the growth rate is higher, but the net increase in area is relatively small and the expansion intensity is weaker, the net increase in area, growth rate and expansion intensity of the Lanxi urban agglomeration as a whole is small, low and the expansion intensity is weaker, the Guanzhong Plain urban agglomeration and Taiyuan urban agglomeration in the midstream region, except for Xi’an and Taiyuan The growth rate of the two urban agglomerations is at a medium level, while the net increase in area of Xi’an, Zhengzhou, Jinan and Qingdao in the Central Plains Urban Agglomeration and Shandong Peninsula Urban Agglomeration in the downstream region is larger and the expansion intensity is stronger, and the growth rate of the two urban agglomerations is higher overall. It indicates that the YB is divided into the east and west along the Hu Huanyong line and the expansion of each urban agglomeration varies greatly, and the economic development level and urban development need to be further balanced.

### Analysis of urban focuses shift trajectory

**Fig 5:**
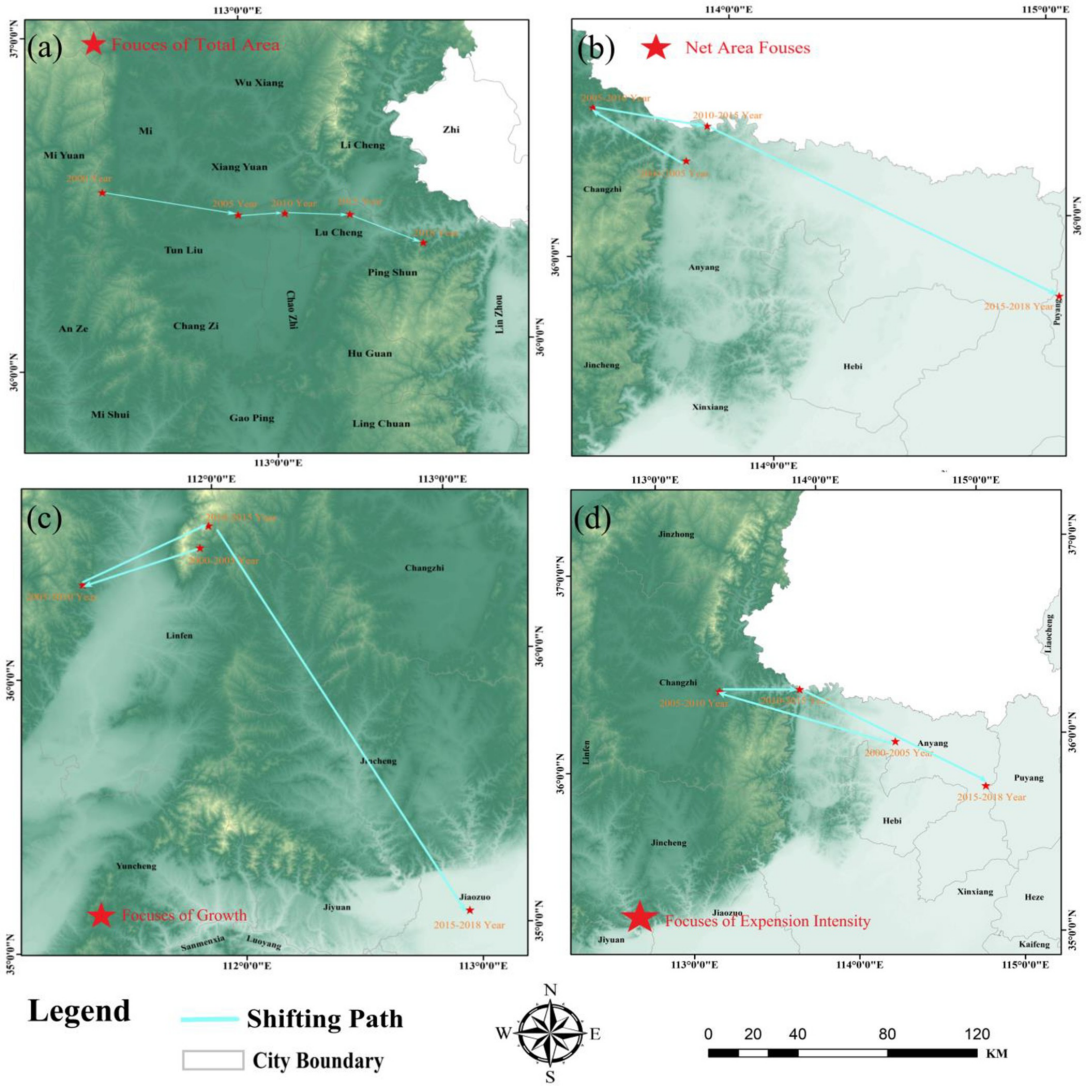
Urban focuses shift trajectory. (a) Focuses transfer of urban build-up area, (b) Net incremented area, (c) Growth rate, (d) expansion intensity in the Yellow River Base from 2000 to 2018.

Generally speaking, the urban expansion in the YB is mainly in the east-west direction, while the upward expansion in the north-south direction is slow. From 2000 to 2018, the focuses of total area, net increase area, growth rate and expansion intensity all shifted significantly to the southeast, indicating that cities in the lower reaches of the YB expanded rapidly, with large net increase area, high expansion intensity and fast expansion speed. The shift results of the focuses in the built-up area of the YB are shown in Fig 5.

From the shift change of area focuses Fig 5(A): During 2000–2018, the focuses of UB areas showed a development trend of "northwest to southeast", and the shift range from east to west was greater than that from north to south. In 2000, the focus of built-up areas was in Qin County, Shanxi Province, and continued to migrate to southeast; while in 2018, the focuses of built-up areas were in Pingshun County, Shanxi Province. We can see that from 2000 to 2018, the built-up area of the YB gradually migrated to the southeast, and the focus was located in the middle and lower reaches, indicating that compared with the upper reaches, the urban expansion area in the middle and lower reaches was large.

From the focuses of net increase area Fig 5(B), during 2000–2018, its UB area showed a development trend of "from northwest to southeast", and the shift range from east to west was greater than that from north to south. From 2000 to 2005, the focus of net increase area was in Anyang City, Henan Province, and shifted northwest to Changzhi City, Shanxi Province from 2005 to 2010. From 2010 to 2015, the focus of net increase area shifted southeast and returned to Anyang City, Henan Province. From 2015 to 2018, the focus of net increase area continued to shift southeast to Puyang City, Henan Province. It shows that during the period from 2000 to 2018, the net area of cities in the upper reaches of the Yellow River was large in early years, but with the development focuses gradually shifting to the southeast, the cities in the lower reaches expanded rapidly and the net area gradually increased.

During 2000 to 2018, from the focuses of growth rate Fig 5(C) we can see that, its UB area showed a development trend of "northwest, then northeast, and then southeast", and the shift range from east to west was greater than that from north to south. From 2000 to 2005, the focus of expansion intensity was in Long’an District, Anyang City, Henan Province, and shifted to Lucheng City, Changzhi City, Shanxi Province from 2005 to 2010. From 2010 to 2015, the focus of expansion intensity shifted to the northeast and returned to Linzhou, Anyang City, Henan Province. From 2015 to 2018, the focus of expansion intensity shifted to the southeast to Neihuang County, Anyang City, Henan Province. It shows that during the period from 2000 to 2018, the focus of expansion intensity gradually shifted to the southeast, locating in the area of lower reaches, and the urban expansion intensity in the area of lower reaches was higher and the city developed rapidly.

During 2000 to 2018, from the focuses of expansion intensity Fig 5(D) we can see that, its UB area showed a development trend of "northwest, then northeast, and then southeast", and the shift range from east to west was greater than that from north to south. From 2000 to 2005, the focus of expansion intensity was in Anyang City, Henan Province. From 2005 to 2010, it shifted northwest to Changzhi City, Shanxi Province; From 2010 to 2015, the focus of expansion intensity shifted to the northeast and returned to Anyang City, Henan Province. From 2015 to 2018, the focus of expansion intensity shifted to the southeast to Puyang City, Henan Province. It shows that during the period from 2000 to 2018, the focus of expansion intensity gradually shifted to the southeast, locating in the area of lower reaches, and the urban expansion intensity in the area of lower reaches was higher and the city developed rapidly.

### Analysis of urban expansion pattern

**Fig 6:**
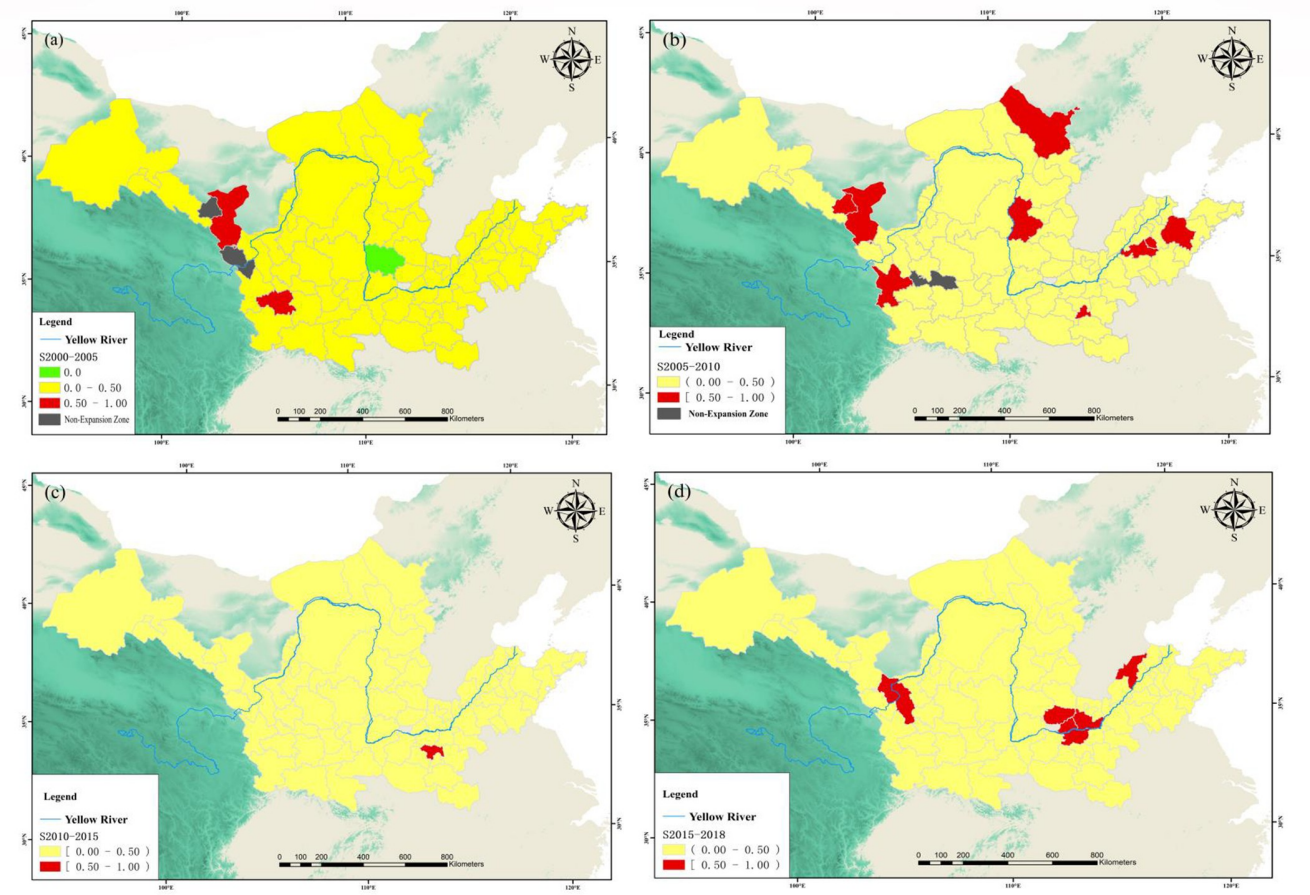
Results of UB area expansion model in the YB from 2000 to 2018. (a) From 2000 to 2005; (b) From 2005 to 2010; (c) From 2010 to 2015; (d) From 2015 to 2018.

According to the data of 78 UB areas in the YB from 2000 to 2018, firstly, the common edge of new UB areas and existing UB areas and the land boundary of new UB areas in four study periods are extracted, and then the common edge index and urban expansion pattern in four study periods are calculated by using the common edge detection method, as shown in Fig 6. When *S*≥0.5, it is filled expansion; When 0<*S*<0.5, it is spreading-type expansion; And when *S* = 0, it indicates that there is no common edge between the land used in the existing built-up area of the city and the land used in the newly added built-up area of the city, which is an enclave expansion.

The expansion mode of UB areas in the YB is shown in **Table 2**. On the whole, a large proportion of spreading-type cities indicate that the urban development in the YB is still dominated by the traditional "urban sprawl" expansion, and has not yet entered the stage of "quality-oriented" urban development and upgrading. In the future development, in order to achieve the high-quality development of the YB, we must take the road of urban expansion with high-quality development as the core. Only relying on the continuous extension of urban development model will not only have a certain impact on ecology and agriculture, but also make the efficiency of urban land use gradually decline.

**Table 2:** Proportion of UB area expansion mode in the YB.

Year	Total number of cities	Number of spreading-type cities	Proportion of spreading-type cities/%	Number of filling-type cities	Proportion of filling-type cities/%	Number of enclave-type cities	Proportion of enclave-type cities/%
2000–2005	78	75	96.15	2	2.56	1	1.28
2005–2010	78	69	88.46	9	11.54	0	0
2010–2015	78	77	98.72	1	1.28	0	0
2015–2018	78	72	92.31	6	7.69	0	0

From 2000 to 2018, the expansion mode of UB areas in the YB was mainly spreading-type, and the proportion of spreading-type expansion mode reached 96.15%, 88.46%, 98.72% and 92.31% respectively in four study periods; The number of filled expansion is small, and the number in four periods is 2, 9, 1 and 6 respectively; The enclave expansion mode appeared only once in the study period, which was Linfen City from 2000 to 2005.

Among them, Zhengzhou City, Xinxiang City, Jiaozuo City, Dezhou City, Jincheng City and Baiyin City mainly expanded their built-up areas from 2000 to 2015. From 2015 to 2018, the expansion mode of built-up areas in six cities changed from spreading to filling, which indicated that with the expansion of UB areas, it gradually shifted to internal filling expansion, paid more attention to transformation and sustainable development, and made efficient use of existing urban space, which was conducive to promoting the development of cities towards compact land use. The expansion mode of built-up areas in Wuwei City and Tianshui City from 2000 to 2005 was filling type, and from 2005 to 2018, it changed from filling type to spreading type; Dingxi City, Jinchang City, Luohe City, Laiwu City, Tai’an City, Weifang City, Ulanqab City and Luliang City showed the expansion mode of "spreading-filling-spreading" from 2000 to 2018, which indicated that the above eight cities were constantly filled and improved in the early cities, and gradually changed into spreading-type expansion mode with the continuous expansion of UB areas.

## Discussions

Urban expansion covers a variety of factors, and the increase of urbanization level and economic development have an important impact on urban expansion. The urban development in the YB is uneven, and the level of economic development varies greatly among cities [3]. Based on the reality, the expansion of cities with developed economy, convenient transportation and high population density is more significant [28–30,36], and the results of this paper found that the difference in expansion between provincial capital cities and non-capital cities, as well as between upper and middle and lower reaches of cities due to economic and social factors, is significant. Furthermore, the sprawl-based expansion pattern of cities in the YB indicates that there is irrational urban expansion in the YB. For this reason, the cities in the YB should consider both the coordinated development of population growth and intensive land use in the YB in future expansions, as well as the continuous optimization of intra-city construction and improvement of land utilization in urban expansions. Therefore, the results of this study suggest that the government should implement a rational and efficient urban planning concept, effectively achieve scientific planning, emphasize both high-quality economic developments rather than simple outward expansion, and consider the ecological environment of the city comprehensively to promote sustainable urban development."

To explore the urban expansion status of the YB, objective, accurate and time-sensitive NTL remote sensing image data are used as the data source, and the spatial and temporal patterns of urban expansion evolution, center of gravity migration and expansion patterns in the YB from 2000 to 2018 are studied by using the center of gravity migration and public edge measurement model. For one hand, it remedies the problem of statistical data’s timeliness difference and inability to visualize, and on the other hand, it provides data support for the high-quality development of the YB, as well as support reference for other basin urban planning and management decisions.

Finally, there are four main types of methods for extracting UB areas: empirical threshold method, mutation detection method, statistical data method and higher resolution image data spatial comparison method, each of which has its own advantages and disadvantages, such as the empirical threshold method is convenient but less accurate, and the image data comparison method is highly accurate but less convenient cross. Consequently, combining the advantages of various extraction methods in future studies will lead to more accurate extraction of UB areas [40].

## Uncertainties

There are also some shortcomings in the research process of this paper. Firstly, in terms of data, DMSP/OLS and NPP/VIIRS lighting data are acquired by different sensors with a spatial resolution of 1 km for the former and 500 m for the latter, and the published DMSP/OLS data span from 1992 to 2013, while the NPP/VIIRS monthly data are updated from 2012, so the separate calibration of the two data The fusion of the two types of data is an important prerequisite for long time scale studies. This study draws on existing research results and fuses them, but there is still room for improvement in the fusion effect. In the future study, it is important to scientifically and accurately fuse the two data based on more accurate separate calibration of the two data.

Besides, urban expansion reflects regional differences, and its expansion differences are influenced by various driving factors. Exploring the expansion of its UB areas and the mechanisms underlying it, as well as the joint action mechanisms of multiple factors, will continue to be the focus of future research, and the driving factors of urban expansion in the basin will continue to be explored in subsequent studies, and the study of its internal coordination and the interaction of its urban system and different urban classes will continue to be strengthened in the future.

## Conclusions

In this paper, the DMSP and NPP NTL data are used to extract the land use information of 78 UB areas in the YB with high accuracy. The urban sprawl measurement model, center of gravity migration model and common edge measurement are used to analyze the UB areas in the YB from 2000 to 2018 in terms of "Spatiotemporal pattern-Center of gravity migrationt-Expansion pattern". The spatial and temporal patterns of built-up areas in the YB are studied in terms of expansion area, growth rate and expansion intensity. On this basis, the spatial distribution characteristics of built-up area changes in the YB are revealed using the center of gravity migration model, which in turn reflects the trend of built-up area expansion, and finally the urban expansion pattern of the YB is explored using the common edge measure.

This study resulted in the following conclusively: the area of UB areas in the YB increased continuously throughout the period from 2000 to 2018, and in the expansion of UB areas, there were significant differences between the upper, middle and lower reaches of the YB, provincial capital cities and non-provincial capital cities, the east and west sides of the Hu Huanyong Line, and various urban agglomerations within the Basin, no matter in terms of net increase in area, expansion intensity and growth rate. During the period from 2000 to 2018, the migration trajectory of the total built-up area, net increase in area, growth rate and expansion intensity showed the development trend of "from northwest to southeast", and the built-up area continued to increase rapidly in the southeast direction, and the gap between the upper, middle and lower reaches further expanded. The expansion of UB areas in the YB is still dominated by sprawl, and the large proportion of sprawl urban expansion indicates that the urban expansion in the YB is still dominated by the traditional "spreading", and has not yet entered the stage of "quality-oriented" urban development. In the future development process, in order to achieve high-quality development in the YB, it is necessary to move towards a healthy track of urban expansion with "high-quality development as the core".
